# RNA N6-methyladenosine demethylase FTO targets MOXD1 promoting the malignant phenotype of gastric cancer

**DOI:** 10.1186/s12876-023-03065-y

**Published:** 2024-01-10

**Authors:** Yuexing Lai, Hairong Dong, Ping Xu, Jing Wang, Wen Feng, Zhenya Zhao, Linyu Sha

**Affiliations:** Department of Gastroenterology, Songjiang Hospital Affiliated to Shanghai Jiaotong University School of Medicine, Shanghai, China

**Keywords:** FTO, MOXD1, Gastric cancer, m6A modification

## Abstract

**Background:**

The m6A modified demethylase FTO affects the progression of gastric cancer (GC), and the role mechanism of FTO in GC is still unclear. We, here, explored the role of FTO and unrevealed the mechanisms of its function in GC.

**Methods:**

The expression and clinical prognosis of FTO in GC were examined via UALCAN and GEPIA online databases. Effect of FTO shRNA on GC cellular malignant phenotype were proved by CCK-8, Transwell, Wound healing assay and Flow cytometric assay. RNA-sequencing data of FTO depleted AGS cells were downloaded to analyze differentially expressed genes of FTO downstream. The GO and KEGG pathway enrichment were performed for the DEGs by DAVID. RT-qPCR and RIP-qPCR assay were applied to verify the MOXD1 mRNA and methylated mRNA in FTO shRNA group. The expression and clinical prognosis of MOXD1 in GC were explored via UALCAN, GEPIA and Kaplan-Meier plotter. The role and mechanism and of MOXD1 in GC cell lines were detected and analyzed.

**Results:**

The expression of FTO was found to be elevated in GC tissues compared with normal tissues, and worse survival were strongly related to high expression of FTO in GC. FTO silencing suppressed the proliferation, migration and promoted apoptosis of GC cells. A total of 5856 DEGs were obtained in between NC and FTO depleted AGS cell groups, and involved in the cancer related pathways. Here, FTO targets MOXD1 mRNA and promotes its expression via m6A methylation. MOXD1 upregulation was associated to poor prognosis of GC. MOXD1 silencing suppressed the malignant phenotype of GC cells. MOXD1 activated cancer -related signaling pathway (MAPK, TGF-β, NOTCH and JAK/STAT).

**Conclusions:**

Our study demonstrated that FTO silencing decreased MOXD1 expression to inhibit the progression of GC via m6A methylation modification. FTO/MOXD1 may be potential targets for the treatment and prognosis of GC.

## Introduction

Gastric cancer is a prevalent malignancy of the digestive system worldwide, and its incidence ranks the first in digestive tract tumor and mortality rate for 25% of all malignant tumor deaths [1, 2]. Due to the lack of specific symptoms and gastroscopy in the early stage, the diagnosis rate of early GC is particularly low and most patients are diagnosed with advanced GC [3]. Nearly 60% of patients undergoing manual surgery will have recurrence or distant metastasis, and the median overall survival (OS) is not more than 12 months [4]. Another reason for the poor prognosis of GC patients is the lack of targeted drugs and the existing targeted drugs have certain limitations. Although trastuzumab combined with chemotherapy can improve the OS of patients, only about 20% of patients have overexpression of HER2, limiting the application of trastuzumab [5]. Therefore, elucidating the molecular mechanism of GC progression is crucial for early diagnosis, development of new therapeutic targets, and better improvement of the survival time of GC patients.

N6-methyladenosine (m6A) is the most prevalent internal RNA modifications in eukaryotes, which is widely present in mRNA, lncRNA, and circular RNA [6, 7]. The proteins involved in the regulation of m6A modification can be roughly divided into three categories: m6A methylases, including METTL3, METTL14, and WTAP; m6A demethylases, including FTO, ALKBH5; m6A recognizes proteins, including YTHDC1/2, YTHDF1/2/3, etc. Dysregulation of m6A modification due to aberrant expression of its regulatory proteins can often be observed in many pathological situations, especially cancer [8, 9]. FTO plays an important regulatory role in acute myeloid leukemia, breast cancer, melanoma, lung cancer and GC [10–12]. The role of FTO in GC has been extensively reported, but its specific mechanism of action remains unclear [13].

DBH-like monooxygenase 1 (MOXD1) belongs to the copper monooxygenase family. Its discovery occurred during a study aimed at identifying genes that exhibit altered expression during aging in human fibroblasts. Clinical databases showed that MOXD1 was upregulated in GBM cells and promoted cancer cell growth [14]. In addition, MOXD1 expression was one of the biomarkers of early gastric cancer [15]. The expression and function of MOXD1 are still unclear.

The objective of this study was to validate the role of FTO in promoting gastric cancer (GC) progression and evaluate the relationship between FTO expression and overall survival (OS) of GC patients. Next, we explored the downstream function mechanism and targets of FTO by RNA-sequencing dataset. Interestingly, MOXD1 was the target gene of FTO. Furthermore, the expression, function, prognostic and mechanism of MOXD1 were investigated in GC patients and cell lines.

## Methods and materials

### Data acquisition

The microarrays data of gene expression profile of NC and FTO depleted AGS cells were obtained from GEO database (https://www.ncbi.nlm.nib.gov/geo/), including 3 NC and 3 FTO shRNA of AGS cells. The pathology data of GC patient was obtained from THE HUMAN PROTEIN ATLAS (http://www.proteinatlas.org/about/download) to obtain the prognostic favorable/unfavorable genes.

### Differentially expressed gene (DEG) and pathway enrichment analysis

First, the RNA-sequencing data of the 3 negative control (NC) group and 3 FTO shRNA groups were normalized, the DEGs were identified by the R package DEseq2, and the threshold of value was set as *P*-value < 0.05 and |log2 (fold change)| ≥ 1.

The DAVID database (https://david.ncifcrf.gov) was utilized to perform GO enrichment analysis and KEGG pathway analysis in order to investigate the functions and potential signaling pathways of DEGs. KEGG pathway enrichment analysis was performed [16, 17]. GO functional enrichment included biological process (BP) enrichment, cellular component (CC) enrichment, molecular function (MF) enrichment. The terms were significant enrichment with *P*-value < 0.05.

### Gene expression profiling interaction analysis

GEPIA database (http://gepia.cancer-pku.cn/index.html) is an online analysis website for dynamic analysis and visualization of TCGA gene expression profile data. The survival plots of FTO high/low groups in in stomach adenocarcinoma (STAD) sample was analyzed based on GEPIA tool. MOXD1 expression levels and survival plots in stomach adenocarcinoma (STAD) sample were analyzed using the GEPIA tool.

### UALCAN analysis

We employed the online analysis tool UALCAN (http://ualcan.path.uab.edu/) to investigate the expression levels of FTO and MOXD1, as well as their correlation with various clinicopathological parameters and prognosis in patients with stomach adenocarcinoma (STAD). UALCAN database is a website tool for online analysis in TCGA, which can analyze the expression profile and clinicopathological parameters of related genes. The FTO/MOXD1 expression lever in STAD tissue type was searched.

### Kaplan-Meier plotter (gastric cancer)

The KM plotter (http://kmplot.com/analysis/) is a public online survival analysis website, whose data are derived from GEO, and TCGA. Currently, the largest datasets include gastric cancer (1440 patients), gene expression and related survival data. The OS, post-progression survival (PPS) and first progression (FP) of MOXD1 in GC was analyzed on KM-plotter online database.

### Cell culture and transfection

The gastric cancer (GC) cell lines, AGS and HBG823, were acquired from the ATCC. The cells were cultured in RPMI-1640 medium supplemented with 10% FBS, 100 μg/mL streptomycin, and 100 U/mL penicillin (Gibco, USA) and were maintained in a humidified atmosphere containing 5% CO2 at 37 °C. The shRNA sequences of FTO and MOXD1 were synthesized by shanghai GenePharma. FTO shRNA1: 5′-GGATGACTCTCATCTCGAA-3′; FTO shRNA2: 5′-GTCACGAATTGCCCGAACA-3′. MOXD1 shRNA: 5′-CCGGCCATACTTTGATCTGGTAAATCTCGAGATTTACCAGATCAAAGTATGGTTTTTG-3′. MOXD1 shRNA was inserted into the pLKO.1 vector. For cell transfection, AGS and HBG823 were transfected by using LipofectamineTM 3000 (Invitrogen, USA).

### RT–qPCR assay

Total RNA was extracted by using TRIzol from AGS and HBG823 cells. Total RNA was synthesized to cDNA by reverse transcription kit. The cDNA was amplified using the AceQ Universal SYBR qPCR Master Mix on an ABI 7500 PCR system for quantitative PCR (qPCR). The primer sequences were as follows: GAPDH-F: 5′-GAAGGTGAAGGTCGGAGTC-3′, GAPDH-R: 5′-GAAGATGGTGATGGGATTT-3′; MOXD1-F: 5′-GCATCAGGCTGCGTCATTTTC-3′, MOXD1-R: 5′- TCGTGTTGTAGCGACTCAG-3′.

### Cell proliferation assay

Cell counting kit-8 was used to detect the cell proliferation. In brief, AGS and HBG823 cells were seeded in 96 well plates after transfection and cultured in an incubator. At 6, 24, 48, and 72 hours, the 96 well plates were removed and added with 10 μL of CCK-8 solution. After 2 h of incubation, the absorbance of OD450 nm was read using a microplate reader.

### Flow cytometric assay

Cell apoptosis was measured using Annexin V-FITC/PI kit. Briefly, AGS and HBG823 cells were collected and washed with PBS after transfection. The cells were then incubated in 500 μl binding buffer for 15 minutes before adding 5 μl of Annexin V-FITC/PI and staining the cells for another 15 minutes. Finally, the percentage of apoptotic cells was determined by flow cytometry analysis.

### Cell migration assay

After transfection for 48 hours, the cells were resuspended in serum-free medium at a concentration of 1 × 10^5 cells/100 μl and seeded into the upper chamber of Transwell chambers placed in a 24-well plate. The lower chamber was filled with 600 μl of medium containing 10% FBS. Transwell chambers were removed after 48 h, stained with 0. 1% crystal violet. After washing with PBS, pictures were taken under a micromicroscope and counted.

For the wound healing assay, AGS and HBG823 cells were seeded in 6-well cell culture plates and allowed to form a monolayer. A 200 μl pipette tip was used to create a scratch on the monolayer of cells, which were then washed with PBS. Cellular migration into the wounded area was observed under a microscope and photographs were taken at 0 and 36 hours after the scratch was made.

### Methylated RNA immunoprecipitation (RIP) PCR assays (MeRIP-PCR)

After RNA extraction from the AGS and HBG823 cells, 200μg of RNA was fragmented to a size of 200 nt using the Fragmentation Kit, and 5% of the sample was used as input. The remaining samples were added with m6A antibody (Abcam, 1:100) and lgG antibody-beads mixture, respectively. After incubation for 2 h at 4 °C, the plates were washed a total of five times using high and low salt wash solutions. The beads were digested with proteinase k digest to release the enriched RNA, which was purified with the use of an RNA purification kit (QIAGEN). RNA was reversed to cDNA using a reverse transcription kit (Novazan) for subsequent quantitative PCR.

### GSEA analysis of MOXD1 related pathways

Based on the median MOXD1 expression, gastric cancer specimens were divided into two groups, including high expression group and low expression group. GSEA was used to find differences in the signature gene sets of MSigDB between high and low groups.

### Statistical analysis

Statistical analyses were performed using SPSS21. Data are presented as mean ± standard deviation (SD), each conducted in triplicate. Student’s t-test was untiled to compare the means with multiple comparisons. A *p*-value of less than 0.05 was considered statistically significant.

## Results

### FTO expression is upregulated in gastric cancer and associated with a poor prognosis

**Fig. 1:**
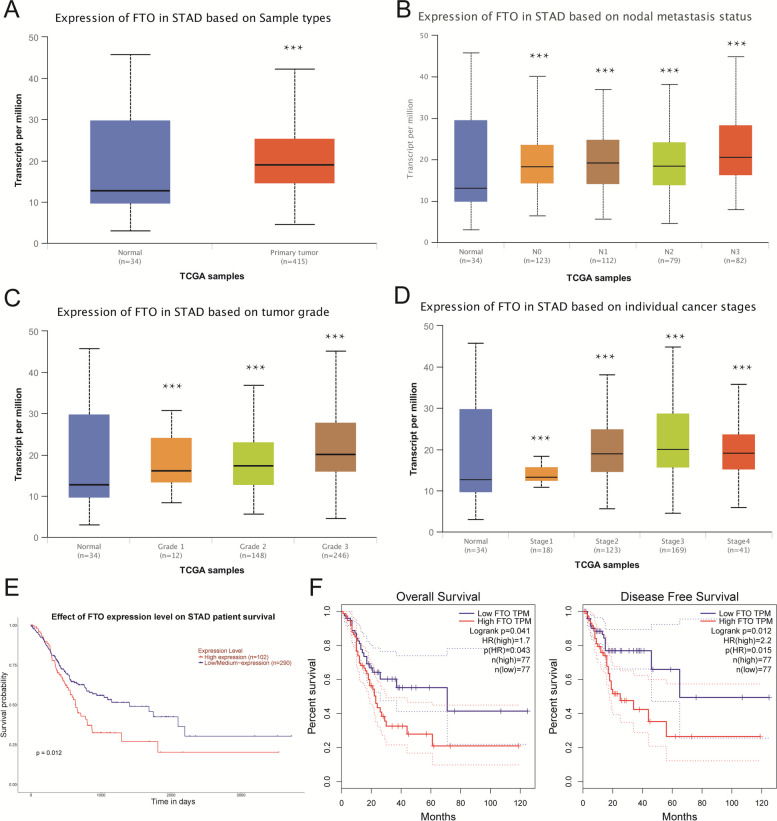
Expression and prognosis value of FTO in STAD. **A** FTO expression in STAD based on Sample types (Normal and Primary tumor). **B** FTO expression in STAD based on nodal metastasis status (Normal, N0, N1, N2, N3). **C** FTO expression in STAD based on tumor grade (Normal, Grade1, Grade2, Grade3). **D** FTO expression in STAD based on individual cancer stages (Normal, Stage1, Stage 2, Stage 3, Stage4). **E** Effect of FTO expression level on STAD patients’ survival. **F** Survival curves of overall survival and disease-free survival of FTO high expression and FTO low expression of STAD

We examined FTO expression in STAD by applying UALCAN online database. FTO expression was higher in Primary tumor group than Normal group (Fig. 1A). FTO expression was significantly higher in all lymph node stage than normal (Fig. 1B). t FTO expression gradually increased with the increase of grade 1- grade 3 (Fig. 1C). FTO expression was higher in the middle and late-stage than the early stages of STAD (Fig. 1D). Above results revealing a potential function for FTO in the development and metastasis of GC.

Next, to explore the significance of FTO expression in GC, we analyzed the correlation between the FTO expression and OS and DFS of STAD patients. Elevated expression of FTO was found to be correlated with a decreased OS in STAD patients by applying UALCAN online database (Fig. 1E). Notably, FTO expression was related to unfavorable outcome of GC patients, and this study revealed that upregulation of FTO was used as marker for worse OS and DFS in STAD patients (Fig. 1F).

### Knockdown of FTO inhibited proliferation, migration and induced apoptosis of GC cells

**Fig. 2:**
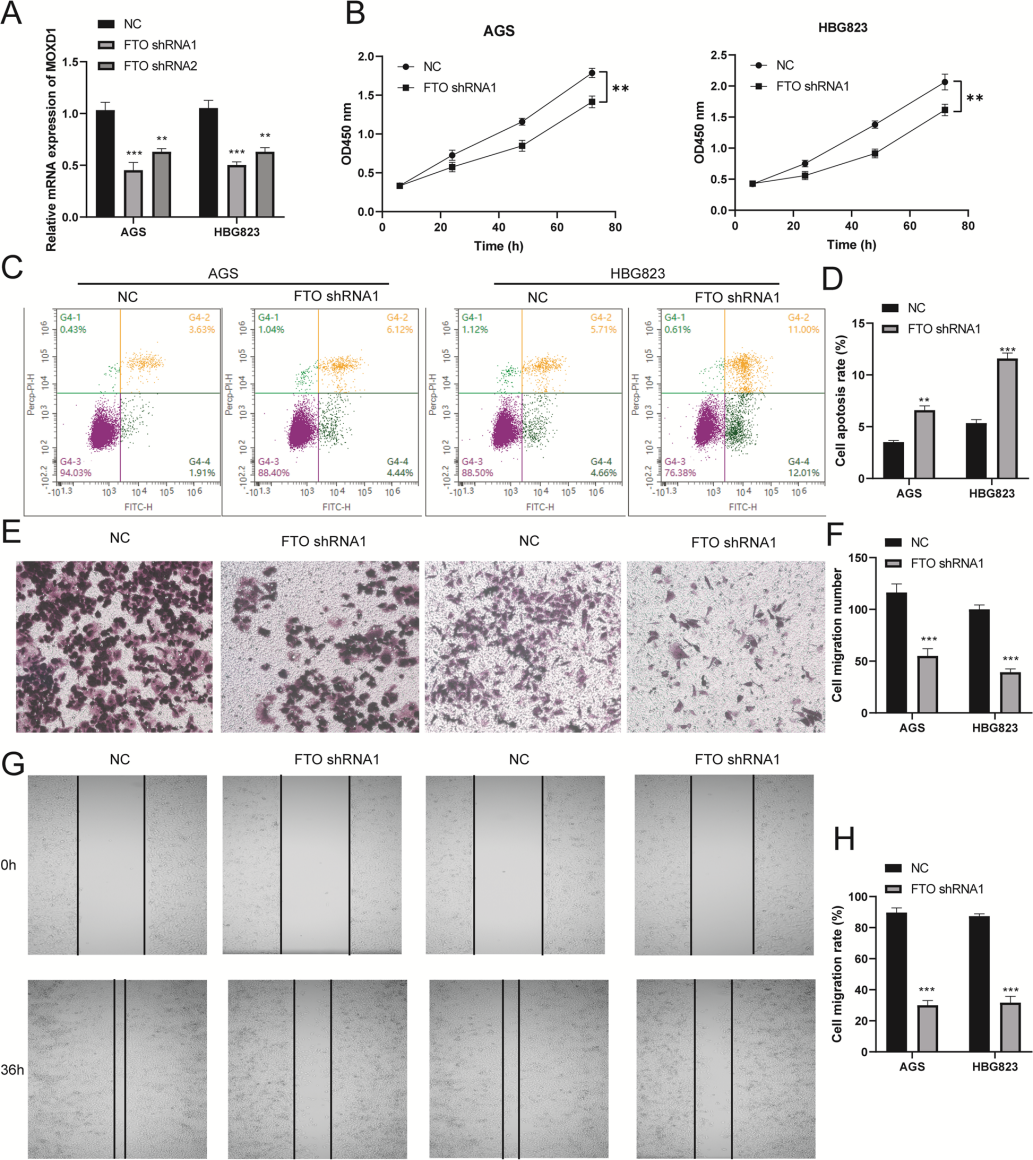
Effects of FTO on the proliferation, migration and apoptosis of AGS and HBG823 cells. **A** FTO expression was analyzed by RT-qPCR assay. **B** CCK-8 assay detected the AGS and HBG823 cell proliferation. **C** A flow cytometry assay detected the apoptosis of AGS and HBG823 cells. **D** Apoptosis rate of AGS and HBG823 cells. **E** Transwell assay and (**G**) A wound healing assay was performed to explore the migration of AGS and HBG823. **F** Cell migration numbers of AGS and HBG823. **H** Cell migration rate of AGS and HBG823

We investigated the role of FTO in GC AGS and HBG823 cell lines. RT–qPCR assays verified that the transfected efficiency of FTO shRNA1, shRNA2 in AGS and HBG823 cells, shRNA1 was most significant (Fig. 2A). CCK-8 assay shown that knockdown of FTO significantly reduced the proliferation of AGS and HBG823 cells (Fig. 2B). Next, knockdown of FTO enhanced the apoptotic rates of AGS and HBG823 cell by flow cytometry assay (Fig. 2C and D). Next, transwell and wound healing assay verified that cell migration was inhibited in the FTO shRNA1 group (Fig. 2E–H).

### Differentially expressed genes and functional enrichment analysis

**Fig. 3:**
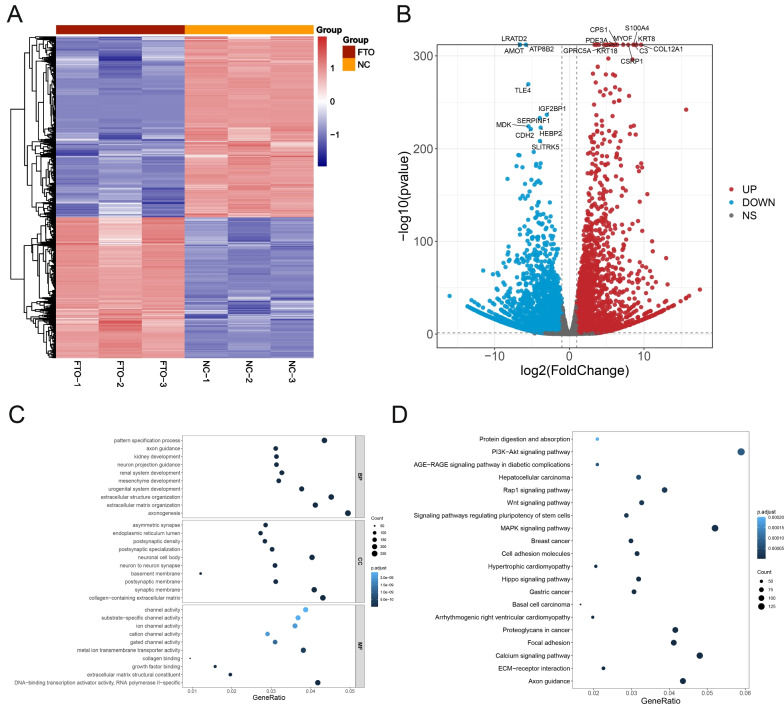
Functional enrichment analysis of DEGs in NC and FTO depleted AGS cells. **A** Heatmap of DEGs in the NC group compared with the FTO depleted AGS groups. **B** Volcano plot showed that upregulated genes (Red) and downregulated genes (Blue) in FTO depleted AGS groups compared with NC group. **C** The top10 enriched biological process (BP), CC and MF of the DEGs. **D** The top20 enriched KEGG pathway of DEGs

To investigate the molecular mechanism underlying how FTO promotes the progression of malignant phenotype of GC, RNA-sequencing data were downloaded to analyze differentially expressed genes of FTO downstream. A total of 5856 DEGs were identified in between NC and FTO depleted AGS cell groups. Heatmap showed that expression of 5856 DEGs in NC and FTO depleted AGS cell (Fig. 3A). Volcano plot showed that 3294 upregulated genes (CPS1, PDE3A, S100A4, MYOF, KRT8, C3, COL12A1, CSRP1, GPRC5A and etc.) and 2562 downregulated genes (LRATD2, AMOT, ATP8B2, TLE4, IGF2BP1, SERPINF1, MDK, CDH2, HEBP2, SLITRK5 and etc.) in FTO depleted AGS groups compared with NC group (Fig. 3B).

To further explore the function of DEGs, we performed GO and KEGG analysis using DAVID. The top 10 enriched terms of BP, CC and MF were shown in Fig. 3C. The terms of pattern specification process, axon guidance, kidney development, extracellular structure organization, extracellular matrix organization of BP, endoplasmic reticulum lumen, basement membrane, synaptic membrane, collagen-containing extracellular matrix of CC, channel activity, ion channel activity, collagen binding, growth factor binding, extracellular matrix structural constituent of MF was enriched in FTO regulated DEGs. The pathways of PI3K-Akt, Rap1, Wnt, MARK, Hippo signaling pathway were enriched in FTO regulated DEGs (Fig. 3D).

### FTO directly targets MOXD1 mRNA and promotes its expression

**Fig. 4:**
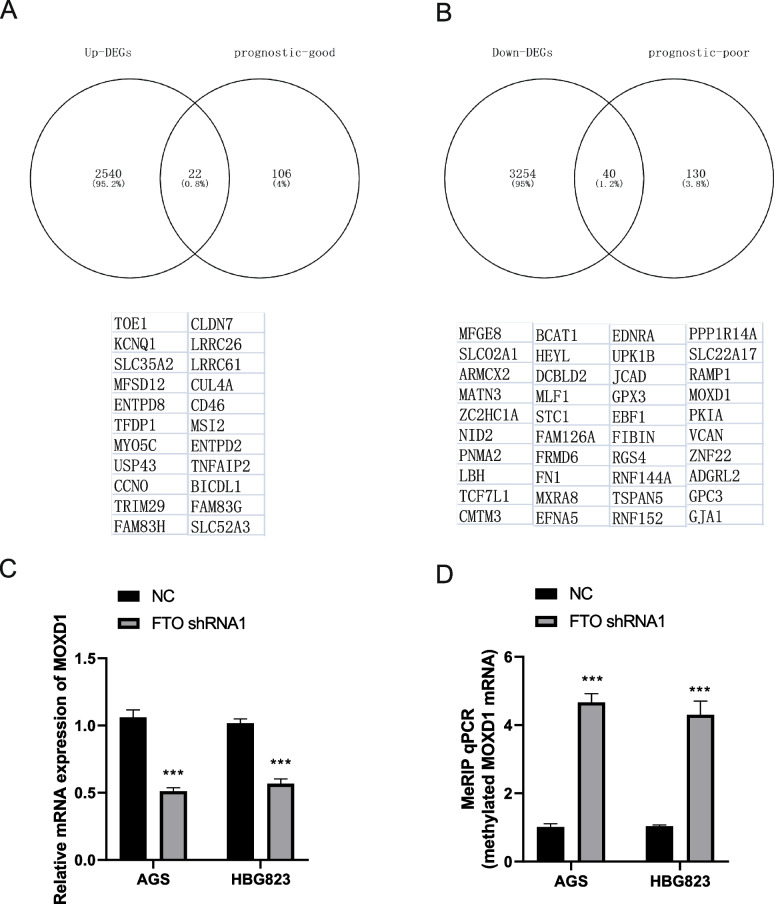
MOXD1 is targeted genes of FTO downstream. **A** Venn diagram showed the overlap of prognostic favorable genes between the up-regulated genes in FTO depleted AGS cells and prognostic favorable genes in GC patients. **B** Venn diagram showed the overlap of prognostic -unfavorable genes between the down-regulated genes in FTO depleted AGS cells and prognostic unfavorable genes in GC patients. **C** RT-qPCR assay was used to verify the MOXD1 mRNA expression in NC and FTO shRNA of AGS and HBG823 cells. **D** m6A methylated mRNA of MOXD1 was detected by RIP-qPCR

To identify the targeted genes of FTO downstream in GC, the pathology data of GC patient was obtained from THE HUMAN PROTEIN ATLAS to analyze the prognostic significance of DEGs in between NC and FTO depleted AGS cells. Next, Venn diagram showed that common 22 prognostic favorable genes between the up-regulated genes in FTO depleted AGS cells and prognostic favorable genes of GC patients, including TOE1, KCNQ1, SLC35A2, MFSD12, ENTPD8, TFDP1, MYO5C, USP43, CCN0, TRIM29, FAM83H, CLDN7, LRRC26, LRRC61, CUL4A, CD46, MSI2, ENTPD2, TNFAIP2, BICDL1, FAM83G, SLC52A3 (Fig. 4A). The common 40 prognostic -unfavorable genes between the down-regulated genes in FTO depleted AGS cells and prognostic unfavorable genes in GC patients, including MOXD1, etc. (Fig. 4B). Further, RT-qPCR assay verified that FTO silencing significantly inhibited the mRNA expression of MOXD1 in AGS and HBG823 cells (Fig. 4C). RIP-qPCR assay found that m6A methylated MOXD1 mRNA level was significantly increased in FTO shRNA group compared with NC of AGS and HBG823 cells (Fig. 4D). Therefore, MOXD1 is directly targeted genes of FTO downstream in GC cells.

### MOXD1 expression was upregulated in STAD sample

**Fig. 5:**
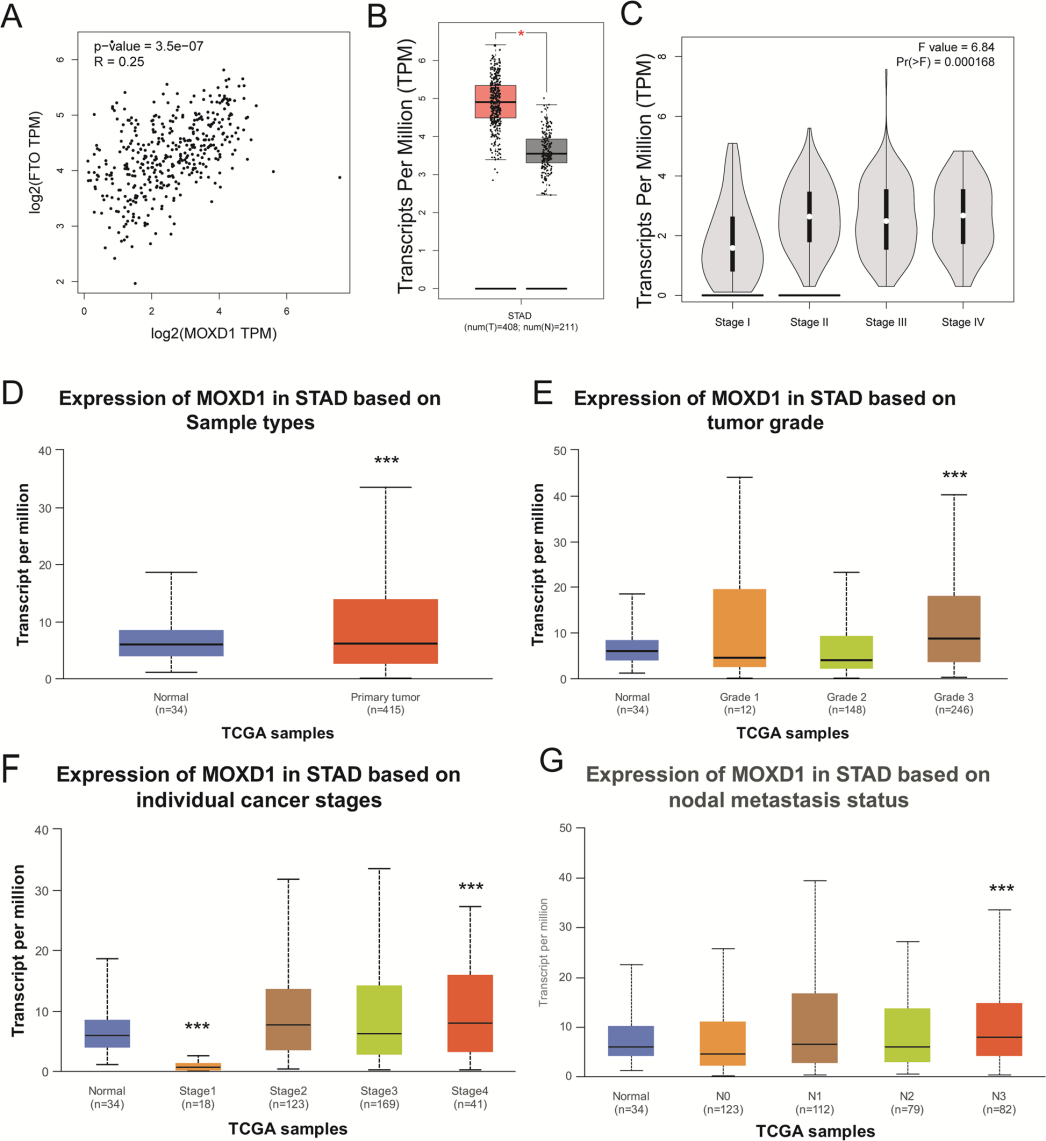
Expression of MOXD1 in STAD. **A** correlation MOXD1 expression and FTO expression in GC. **B** MOXD1 expression in STAD tumor and normal group. **C** Expression of MOXD1 in Stage I-IV of STAD. **D** MOXD1 expression in STAD based on Sample types. **E** Expression of MOXD1 in STAD based on tumor grade. **F** MOXD1 expression in STAD based on individual cancer stages. **G** MOXD1 expression in STAD based on nodal metastasis status

Next, correlation FTO expression and MOXD1 expression in STAD were analyzed in TCGA-STAD dataset based on GEPIA. As shown in Fig. 5A, MOXD1 expression positively correlated with FTO expression in STAD. MOXD1 expression was increased in STAD tumor (Fig. 5B) and STAD stage II, III, and IV compared with Stage I (Fig. 5C). We further analyzed MOXD1 expression in STAD based on Sample types, tumor grade, individual cancer stages, nodal metastasis status by applying UALCAN online database. MOXD1 expression was higher in Primary tumor group than Normal group (Fig. 5D). FTO expression in tumor stage samples was significantly higher in Grade 3 compared with Normal and lower in Grade 1 and Grade 2 compared with Normal (Fig. 5E). MOXD1 expression was decreased in Stage1 and increased Stage 2–4 compared with Normal (Fig. 5F). MOXD1 expression was significantly higher in the middle and late-N3 cancers than the N0 of nodal metastasis status (Fig. 5G). Above results revealing a potential function for MOXD1 in GC progression and metastasis.

### Increased MOXD1 expression associated with poor prognosis of gastric cancer patients

**Fig. 6:**
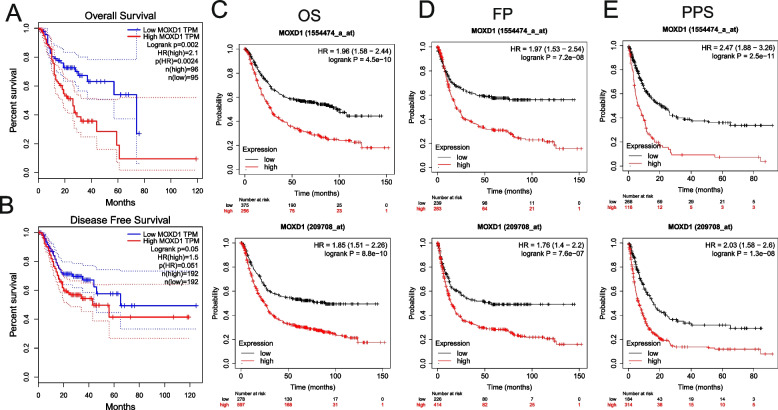
Kaplan-Meier survival curves were used to compare the high and low expression of MOXD1 in gastric cancer. Survival curves of OS (**A**) and DFS (**B**) in the TCGA-STAD dataset. Survival curves of OS (**C**), FP (**D**) and PPS (**E**) in the gastric cancer cohort (209,708 and 1,554,474) in Kaplan-Meier plotter databases

Subsequently, we evaluated the expression of MOXD1 and its correlation with patient prognosis by utilizing Kaplan-Meier survival curves. The aim was to investigate whether MOXD1 may serve as a potential prognostic biomarker for GC. Notably, our findings demonstrated that FTO expression was related to an unfavorable outcome in GC, while increased MOXD1 expression was significantly associated with a poorer prognosis in the GC cohort (Fig. 6).

### MOXD1 silencing suppressed cell proliferation, migration and promoted cell apoptosis in GC cell lines

**Fig. 7:**
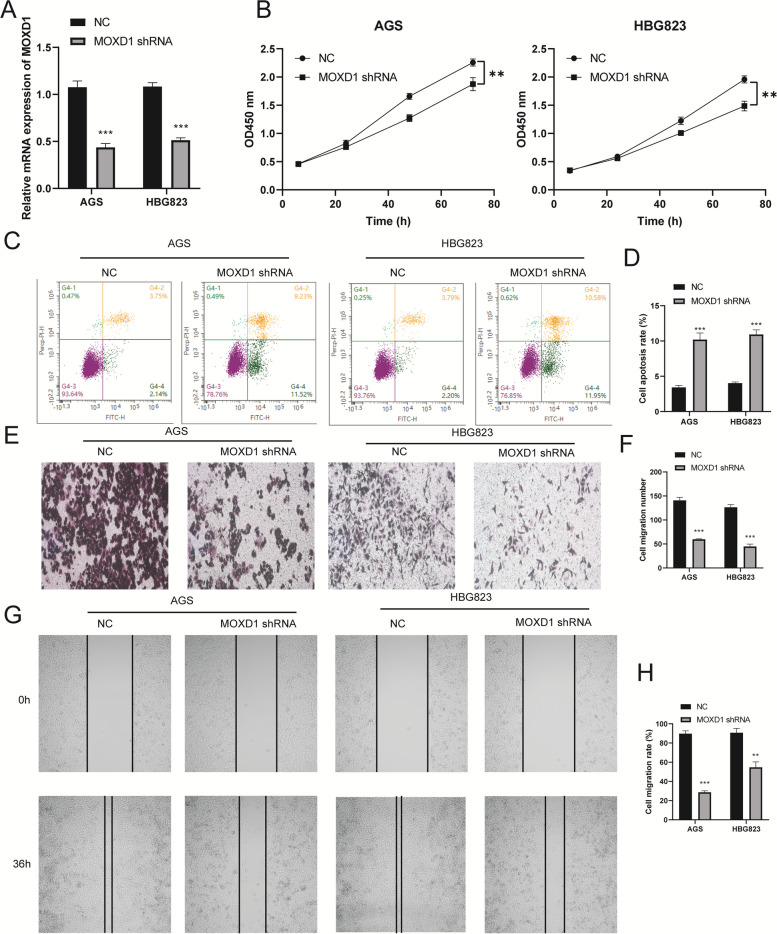
Effects of MOXD1 on the proliferation, migration and apoptosis of AGS and HBG823 cells. **A** MOXD1 expression was analyzed by RT-qPCR assay. **B** CCK-8 assay detected the AGS and HBG823 cell proliferation. **C** A flow cytometry assay detected the apoptosis of AGS and HBG823 cells. **D** Apoptosis rate of AGS and HBG823 cells. **E** Transwell assay and (**G**) A wound healing assay were performed to explore the migration of AGS and HBG823. **F** Cell migration numbers of AGS and HBG823. **H** Cell migration rate of AGS and HBG823

In order to scrutinize the functions of MOXD1 in GC, stable MOXD1 knockdown constructs were introduced into AGS and HBG823 cells via lentivirus-mediated short hairpin RNAs (MOXD1 shRNA). The transfection efficiency of MOXD1 shRNA was validated by RT-qPCR assays conducted in AGS and HBG823 cells (Fig. 7A). CCK-8 assay results revealed that the proliferation rate of both AGS and HBG823 cells was significantly inhibited upon MOXD1 knockdown, indicating a potential role of MOXD1 in promoting cell proliferation (Fig. 7B). To further investigate the impact of MOXD1 on apoptosis in these cells, flow cytometry analysis was performed. Results indicated that MOXD1 knockdown increased the rate of apoptotic cells overall (Fig. 7C and D). Trans well and wound healing assays were also conducted to determine any changes in cell migration upon MOXD1 knockdown. Compared with the control group, cells with MOXD1 knockdown exhibited reduced migration rates (Fig. 7E–H). Taken together, these findings suggest that suppression of MOXD1 can potentially reduce cell proliferation and migration while inducing apoptosis in GC cells.

### Molecular basis for the role of MOXD1 in GC cells

**Fig. 8:**
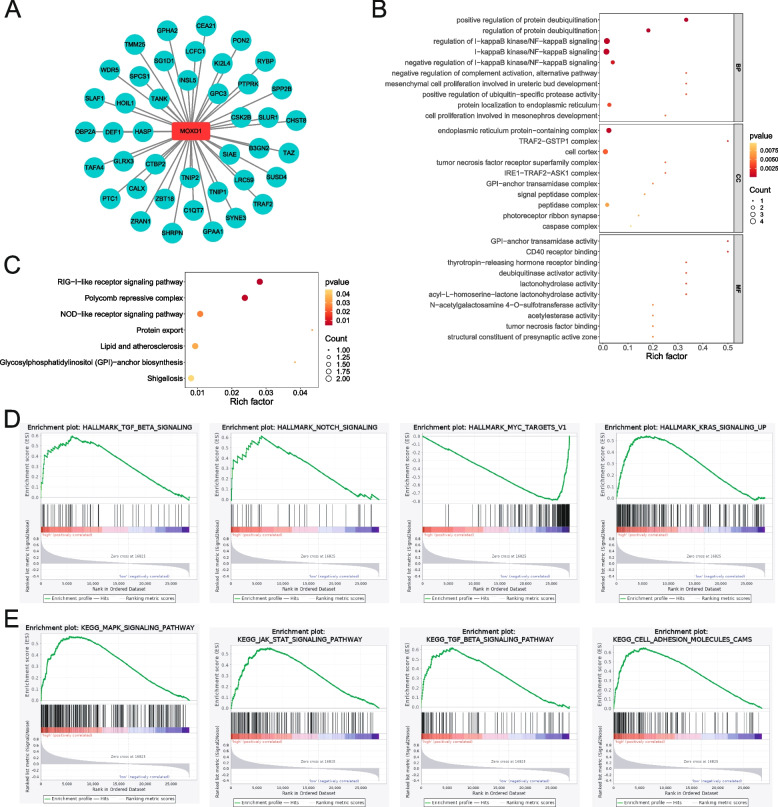
Molecular basis for role of MOXD1 in GC. **A** MOXD1 interacting proteins was predicted by online database (http://www.hitpredict.org/). **B** GO function enrichment analysis. **C** KEGG pathway enrichment analysis. **D** HALLAMARKS pathway of MOXD1 was enriched by GSEA. **E** KEGG signaling pathway of MOXD1 was enriched by GSEA

Thereafter, the molecular basis for the role of MOXD1 in GC was explored through GO and KEGG function enrichment analysis and GSEA analysis. MOXD1 interacting proteins were predicted, including TAZ, PTC1, TAFA4, ZRAN1, SHRPN, GPAA1, TRAF2 and etc. (Fig. 8A). GO function enrichment analysis revealed that these proteins were involved in NF-KappaB signaling, positive regulation of protein deubiquitylation, tumor necrosis factor binding, and etc. (Fig. 8B). KEGG signaling pathway enrichment analysis revealed that these proteins were including RIG-I-like receptor signaling pathway, Polycomb repressive complex, NOD-like receptor signaling pathway and etc. (Fig. 8C). We used hallmarks pathways for GSEA enrichment analysis and found high expression of MOXD1 enriched in TGF-BETA, NOTCH, MYC-targets, KRAS signaling pathway, low expression of MOXD1 enriched in MYC targets (Fig. 8D). KEGG signaling pathways was involved in MAPK, JAK STAT, TGF-beta signaling pathway and Cell adhesion molecules (Fig. 8E). MAPK signaling pathway, TGF-BETA signaling pathway play important in the proliferation, migration, invasion and apoptosis of cancer cells. Therefore, MOXD1 promoted cell proliferation, migration, invasion through activating MAPK signaling pathway etc.

## Discussion

The results of this study revealed that increased expression of FTO was linked to unfavorable prognosis among GC patients. FTO silencing decreased proliferation, migration and induced apoptosis of GC cells. RNA-sequencing revealed that MOXD1 was targeted gene of FTO downstream. Additionally, GC tissues exhibited elevated levels of MOXD1 expression and increased expression of MOXD1 was found to be correlated with reduced OS, DFS, FP, and PFS among GC patients, indicating that MOXD1 promoted the progression of GC. Moreover, FTO regulated MOXD1 expression by m6A modification promote the progression of GC.

FTO was initially reported to be closely related to obesity diseases [18]. Recently, FTO was found to be an N6-methyladenosine demethylating enzyme, which plays an important role in biological process, including cell proliferation, apoptosis, and differentiation [19]. Recent studies have found that FTO is involved in the occurrence and development of various cancer, such as breast cancer, endometrial cancer, thyroid cancer, colorectal cancer and pancreatic cancer [20–22]. However, there are few studies on FTO in GC. Previous study has demonstrated that FTO expression plays a crucial role in the development of GC and could serve as an important molecular marker for both diagnosis and prognostic evaluation in patients with GC [23]. We examined FTO expression in STAD based on Sample types by applying UALCAN online database. There results were consistent with precious studies [24], which reveals high expression of FTO promotes the development and metastasis of GC and associated with a poor prognosis of GC patients.

FTO serve as a demethylase which play an important role in tumorigenesis, metastasis and drug resistance by reducing the m6A methylation level of oncogenes and tumor suppressor genes [25]. FTO functions by demethylating m6A modifications of HOXB13 mRNA, thereby promoting metastasis of endometrial cancer through activation of the WNT signaling pathway [26]. FTO is able to inhibit the occurrence of papillary thyroid carcinoma by downregulating SLC7A11 in m6A independently [27]. Previous study found that FTO, an N6 methyl adenosine demethylase, has been found to facilitate the growth and metastasis of GC through its ability to modify caveolin-1 with m6A and regulate mitochondrial dynamics through metabolic pathways [13]. To further explore the molecular mechanism involved in FTO promoting the progression of malignant phenotype of GC, RNA-sequencing data were downloaded to analyze differentially expressed genes of FTO downstream. Three thousand two hundred ninety-four upregulated genes (CPS1, PDE3A, S100A4, MYOF, KRT8, C3, COL12A1, CSRP1, GPRC5A and etc.) and 2562 downregulated genes (LRATD2, AMOT, ATP8B2, TLE4, IGF2BP1, SERPINF1, MDK, CDH2, HEBP2, SLITRK5 and etc.) were identified in FTO depleted AGS groups compared with NC group. Functional, the terms of cancer cell growth and metastasis related was enriched in FTO regulated DEGs. The pathways of PI3K-Akt signaling pathway and etc. were enriched in FTO regulated DEGs. The pathways play an important role in development of GC [28, 29]. Next, the prognosis value of DEG in GC were analyzed. Further, RT-qPCR assay verified that FTO silencing significantly inhibited the mRNA expression of MOXD1 in AGS and HBG823 cells. RIP-qPCR assay found that m6A methylated MOXD1 mRNA level was significantly increased in FTO shRNA group compared with NC of AGS and HBG823 cells. Therefore, MOXD1 is directly targeted genes of FTO downstream in GC cells.

MOXD1 gene is a member of the copper/ascorbic acid-dependent monooxygenase family. This enzyme is related to some important biological functions of organisms [30]. When cells grow normally, the gene is dormant or only participates in the regulation of some important physiological functions [31]. Once normal cells become malignant due to various factors, abnormal expression will occur to promote the occurrence and development of tumors [32]. Suppression of MOXD1 through knockdown techniques was observed to hinder the proliferation of osteosarcoma cells and impede growth of xenograft tumors by inducing apoptosis [33]. The expression of MOXD1 in GC tumor has not been reported, and the role of MOXD1 in GC has not been studies. This study demonstrated that heightened MOXD1 expression is associated with decreased overall survival rates in patients with gastric cancer. The functional analysis showed that knockdown of MOXD1 reduced cell proliferation and migration while promoting apoptosis in GC cells. In terms of mechanism, MOXD1 activated TGF-BETA signaling, NOTCH signaling, KRAS signaling, MAPK signaling pathway, JAK/STAT signaling pathway. These signaling pathways promoted cancer cells proliferation, migration, and invasion.

In conclusion, our study suggested that the key demethylase of m6A FTO promoted the malignant phenotype of GC cells via regulating the oncogene related pathways. In terms of mechanism. FTO improved the degradation of MOXD1 mRNA via m6A modification. The elevated expression of MOXD1 was found to be linked with unfavorable prognoses in GC, as well as the promotion of malignant phenotype in GC cells. Collectively, our findings provided a new direction of the pathogenesis of GC and a new target and theoretical basis for the treatment strategy of GC.

## Data Availability

The datasets GSE26309 is available in the GEO database (http://www.ncbi.nlm.nij.gov/geo). All data can be accessed by contacting the corresponding author.
